# CD8 ^+^ T cell response to adenovirusvaccination and subsequent suppression of tumor growth: modeling, simulation and analysis

**DOI:** 10.1186/s12918-015-0168-9

**Published:** 2015-06-06

**Authors:** Qing Wang, David J Klinke, Zhijun Wang

**Affiliations:** Department of Computer Sciences, Mathematics, and Engineering, Shepherd University, Shepherdstown, 25443 WV USA; Department of Chemical Engineering and Mary Babb Randolph Cancer Center, West Virginia University, Morgantown, 25606 WV USA; Department of Microbiology, Immunology, & Cell Biology, West Virginia University, Morgantown, 25606 WV USA

**Keywords:** Adenovirus, Vaccination, Modeling, Impulsive ordinary differential equation

## Abstract

**Background:**

Using immune checkpoint modulators in the clinic to increase the number and activity of cytotoxic T lymphocytes that recognize tumor antigens can prolong survival for metastatic melanoma. Yet, only a fraction of the patient population receives clinical benefit. In short, these clinical trials demonstrate proof-of-principle but optimizing the specific therapeutic strategies remains a challenge. In many fields, CAD (computer-aided design) is a tool used to optimize integrated system behavior using a mechanistic model that is based upon knowledge of constitutive elements. The objective of this study was to develop a predictive simulation platform for optimizing anti-tumor immunity using different treatment strategies.

**Methods:**

To better understand the therapeutic role that cytotoxic CD8 ^+^ T cells can play in controlling tumor growth, we developed a multi-scale mechanistic model of the biology using impulsive differential equations and calibrated it to a self-consistent data set.

**Results:**

The multi-scale model captures the activation and differentiation of naïve CD8 ^+^ T cells into effector cytotoxic T cells in the lymph node following adenovirus-mediated vaccination against a tumor antigen, the trafficking of the resulting cytotoxic T cells into blood and tumor microenvironment, the production of cytokines within the tumor microenvironment, and the interactions between tumor cells, T cells and cytokines that control tumor growth. The calibrated model captures the modest suppression of tumor cell growth observed in the B16F10 model, a transplantable mouse model for metastatic melanoma, and was used to explore the impact of multiple vaccinations on controlling tumor growth.

**Conclusions:**

Using the calibrated mechanistic model, we found that the cytotoxic CD8 ^+^ T cell response was prolonged by multiple adenovirus vaccinations. However, the strength of the immune response cannot be improved enough by multiple adenovirus vaccinations to reduce tumor burden if the cytotoxic activity or local proliferation of cytotoxic T cells in response to tumor antigens is not greatly enhanced. Overall, this study illustrates how mechanistic models can be used for in silico screening of the optimal therapeutic dosage and timing in cancer treatment.

## Background

Cytotoxic CD8 ^+^ T cells are important effectors in the adaptive immune response against intracellular pathogens and play an important role in immunosurveillance against malignancy [1, 2]. Modulating an immune checkpoint to increase cytotoxic T lymphocytes (CTLs) that target malignant cells can cure patients of metastatic melanoma [3, 4]. While this clinical success demonstrates proof-of-principle, the clinical response is limited to a subset of patients. Yet, these results encourage alternative approaches to direct host immunity against tumors, including adoptive transfer of autologous T cells extracted from a patient’s own tumor (e.g., [5]), engineering of T cell receptors to recognize tumor antigens (e.g., [6, 7]), or vaccination against tumor antigens (e.g., [8-11]). Cancer vaccines based on patient-specific material is attractive as it would enable personalized treatments that enhance CTL response to the specific antigens expressed by a patient’s tumor [12]. One approach is to use adenoviruses that were initially developed as vehicles for gene therapy. Attempts to replace missing or faulty genes by adenoviral gene transfer were largely unsuccessful in experimental animals and human volunteers alike due to innate and adaptive immune responses induced by the adenoviral antigens ([13]). Replication-deficient adenovirus vectors have been pursued as vaccine carriers in the clinic as they showed high efficiency in some rodent and simian preclinical models [13, 14]. The profile of the immune response elicited by adenovirus vaccines against tumor antigens in murine models was investigated by some research groups (see [15-17]). While the approach seems promising, the results are suboptimal as similarly observed for the immune checkpoint modulators. In exploring one treatment variation, sequential treatments involving adenovirus and oncolytic viruses may lead to improved antitumor response [18]. However a more systematic approach to explore treatment variants may be helpful to improve overall response.

As illustrated by [19-22], a variety of mathematical models based on ordinary differential equations (ODEs) have been developed to better understand cancer progression and response to immunotherapy in the last couple of decades. Early work employed Lotka-Volterra equations to describe the interactions between tumor and the immune system where effector cells acted as predators and tumor cells as prey ([21, 23]). The immune surveillance phenomena was described qualitatively in [23] where low doses of tumor cells can escape immune defenses and grow into a larger tumor whereas larger doses of tumor cells are eliminated. The simple predator-prey model was generalized by Kirschner [21, 24], de Pillis et al. [25], Eftimie et al. [26], Wilson and Levy [27], and other researchers where different components of the immune system, such as particular cytokines or natural killer cells, were introduced into the model depending on different cancer treatment strategies. The effect of time delay in the immune response was considered in [20, 28, 29] where authors found that impact of time delay on tumor growth is almost negligible.

Mathematical modeling and computer simulations can be powerful tools in optimizing therapeutic strategies. Mathematical modeling and simulations can be used to screen in silico parameter regions that seem most promising for optimal timing and dosage of therapy and clinical trials can be focused on those regions [30-32]. In [33], the authors explore how the timing of oral insulin delivery and immunomodulatory drugs can be optimized for maximum effect. Moreover, an in silico approach can suggest targeted experiments and then minimize the number of needed experiments [34]. It can also be applied to combine in a virtual way different modes of actions that are well characterized in isolation, such as immunotherapy and chemotherapy, and see how they may be combined to maximum benefit. For instance, Eftimie et al. explored how vaccination using two different viruses that carry the same tumor antigen achieves a greater therapeutic response than if one virus is used alone [26]. In this paper, we use simulations to investigate the impact of multiple adenovirus vaccinations on CD8 ^+^ T cell proliferation and recruitment to the tumor microenvironment and to identify important parameter ranges that control tumor growth through vaccination-induced anti-tumor immunity.

The structure of this paper is as follows. First, we present a multi-scale mechanistic model of anti-tumor immunity and tumor growth based on a set of coupled impulsive ODEs. Second, we describe how we calibrated the parameters of the model against published experimental data using a genetic algorithm. Next we investigate the stability of tumor-free and high tumor equilibria based on the linearized system. Finally, we used the simulation platform to explore the impact of multiple adenovirus vaccinations on T cell proliferation and recruitment to the tumor microenvironment to control tumor growth.

## Methods

Here, we developed a multi-scale impulsive ODE model based on our mechanistic understanding of underlying biology and calibrated the model using existing experimental data. This multi-scale mathematical model represents the cytotoxic T cell response to adenovirus vaccination against a tumor antigen and subsequent control of the growth of B16F10 tumors. For the reported experiments, the B16F10 cell line was purchased from American Tissue Culture Collection (ATCC, Bethesda, MD). Numerical solutions of the model were obtained using simulators generated by C Sharp. Simulations start on day 0, the time of tumor implantation and conclude on day 49. At the initial time point, we assume that there is no activated tumor specific effector T cells present in the blood and at the site of the tumor. A genetic algorithm was used to find parameter sets that closely match the experimental data [15, 16]. Each parameter set was modeled using an individual chromosome in order to apply the algorithm to search in the parameter space. For each generation, the impulsive ODE set was solved using the Runge-Kutta method of order four for each parameter set. The fitness function value, or variance, was calculated using a linear combination of sum of error squared and sum of differences between slopes of lines of experimental data and corresponding model predictions. The calibrated mechanistic model was then used to investigate the long-term behavior through stability analysis. Finally, we used the calibrated model to explore the impact of multiple vaccinations on tumor growth to improve anti-tumor immunity, a scenario that is difficult to test experimentally using pre-clinical mouse models but could be potentially used in the clinic to treat patients. Details of model development, parameter calibration, stability analysis, and numerical simulations of multiple vaccinations are described in the following sections.

## Results

### A multi-scale model of CD8 ^+^ T cell control of tumor growth

**Fig. 1:**
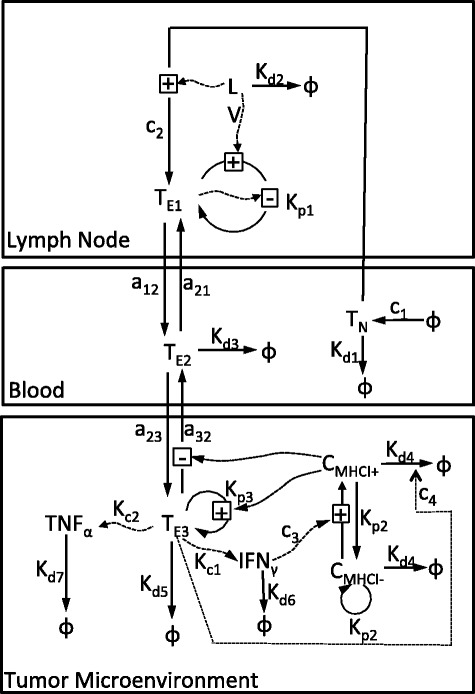
The mechanics of the interactions of three compartments. Naïve CD8 ^+^ T cells (*T*_*N*_) are activated and become CD8 ^+^ T effectors (*T*_*E*1_) when they encounter antigen introduced by the adenovirus (LV) in the lymph node. Once activated from naïve cells, effector CD8 ^+^ T cells circulate within the blood (*T*_*E*2_) and enter tumor microenvironment (*T*_*E*3_) where they are retained upon recognition of the corresponding tumor-associated antigen. Effector CD8 ^+^ T cells secrete Interferon gamma (IFN_*γ*_) and Tumor Necrosis Factor alpha (TNF_*α*_), which assist with the CD8 ^+^ T cell-directed lysis of tumor cells ($C_{\text {MHCI}^{+}}$ and $C_{\text {MHCI}^{-}}$) through increased presentation of tumor-associated antigens by Major Histocompatibility Complex protein class I (MHCI)
- **Effector CD8**^**+**^** T cells in lymph node (*T***_***E*1**_**, units: cells per mm**^**3**^**)**. The increase in the rate of concentration of effector CD8 ^+^ T cells in the lymph node due to activation of naïve CD8 ^+^ T cells from the blood stream is given by $ c_{2} \frac {T_{N}Vol_{b}}{Vol_{\textit {ln}}}\frac {\text {LV}}{\text {LV}+\gamma }$, where *Vol*_*b*_=1.4∗10^3^*mm*^3^ is the volume of the blood compartment ([39]) and *Vol*_*ln*_=0.25 *mm*^3^ is the volume of the lymph node compartment ([40]). We assume that the natural death of effector *T* cells in the lymph node is negligible. Effector CD8 ^+^ T cells in the lymph node proliferate at a rate proportional to *T*_*E*1_, a saturable adenovirus-induced antigen term defined by $\frac {\text {LV}}{\text {LV}+\gamma }$, and an immune checkpoint term defined by $\frac {\alpha }{\alpha +T^{2}_{E1}}$, where *α* is the square root of the saturation constant of *T*_*E*1_. We also assume that influx rate of effector CD8 ^+^ T cells from blood to lymph node is $a_{21}\frac {T_{E2}Vol_{b}}{Vol_{\textit {ln}}}$ and *a*_12_·*T*_*E*1_ is the efflux rate.
- **Adenovirus in lymph node (LV, units: Relative Light Units (RLU) per mm**^**3**^**)**. Since the adenovirus used in the calibration experiments are replicate-defective and include a GFP expression plasmid, we assume an exponential decay model for LV. We also used a difference equation $\Delta \text {LV}(t)=\text {LV}(t^{+})-\text {LV}(t^{-})=\text {LV}_{k}$ to reflect the abrupt change of the concentration of adenovirus during vaccination at time *t*_*k*_, where LV_*k*_ represents the dosage of vaccination at *t*_*k*_ with *k*=1,2,…,*n*.
- **Effector CD8**^**+**^** T cells in blood (*T***_***E*2**_**, units: cells per mm**^**3**^**)**. We assume the effector CD8 ^+^ T cells die naturally at a rate *k*_*d*3_*T*_*E*2_ in blood. The influx rate of effector CD8 ^+^ T cells from lymph node to blood is equal to $a_{12}\frac {T_{E1}Vol_{\textit {ln}}}{Vol_{b}}$ and the efflux rate of effector CD8 ^+^ T cells from blood to lymph node is equal to *a*_21_*T*_*E*2_. The influx rate of CD8 ^+^ T effectors from the tumor to blood is $a_{32}\frac {C_{MHCI^{-}}}{\epsilon +C(t)}\frac {T_{E3}Vol_{t}}{Vol_{b}}$ and the efflux rate of CD8 ^+^ T effectors from blood to tumor is *a*_23_*T*_*E*2_, where $C(t)=C_{MHCI^{-}}+C_{MHCI^{+}}\phantom {\dot {i}\!}$ is the number of tumor cells, $C_{MHCI^{+}}\phantom {\dot {i}\!}$ is the number of major histocompatibility complex (MHC) class I positive tumor cells, $\phantom {\dot {i}\!}C_{MHCI^{-}}$ is the number of MHC class I negative tumor cells, and *ε* is a small positive constant representing a small volume of tissue that excludes tumor and effector CD8 ^+^ T cells in the tumor compartment.
- **MHC class I positive tumor cells (**$\phantom {\dot {i}\!}C_{MHCI^{+}}$**, units: cell number)**. MHC class I positive tumor cells are converted from MHC class I negative tumor cells ($C_{MHCI^{-}}\phantom {\dot {i}\!}$) with the assistance of Interferon *γ* (IFN*γ*) at a rate $c_{3}\frac {\text {IFN}{\gamma }}{k_{1} +\text {IFN}{\gamma }}C_{MHCI^{-}}$ and the effector CD8 ^+^ T cell-mediated MHC class I positive tumor cells death rate is $c_{4} T_{E3}\frac {C_{MHCI^{+}}}{\epsilon +C(t)}\phantom {\dot {i}\!}$. We assume that the dilution rate of MHC class I positive tumor cells due to proliferation is $k_{p2} C_{MHCI^{+}}\phantom {\dot {i}\!}$. The natural death rate of MHC class I positive tumor cells is assumed to be $k_{d4}C_{MHCI^{+}}\phantom {\dot {i}\!}$.
- **MHC class I negative tumor cells (**$\phantom {\dot {i}\!}C_{MHCI^{-}}$**, units: cell number)**. MHC class I negative tumor cells are converted to MHC class I positive tumor cells with the assistance of Interferon gamma (IFN_*γ*_) at a rate $c_{3}\frac {\text {IFN}{\gamma }}{k_{1} +\text {IFN}{\gamma }}C_{MHCI^{-}}$. We assume that the proliferation rate of MHC class I positive tumor cells is equal to $2k_{p2} C_{MHCI^{+}}\phantom {\dot {i}\!}$. As MHC class I positive tumor cells proliferate, they lose MHC class I expression and become MHC class I negative cells. A logistic growth pattern is assumed for the number of MHC class I negative tumor cells in the absence of vaccination treatment.
- **Effector CD8**^**+**^** T cells in tumor microenvironment (*T***_***E*3**_**, units: cells per mm**^**3**^**)**. We assume that effector CD8 ^+^ T cells can proliferate locally upon recognition of the corresponding tumor antigen presented by MHCI positive tumor cells at a saturable rate equal to $k_{p3}\frac {C_{MHCI^{+}}}{\epsilon +C(t)}T_{E3}\phantom {\dot {i}\!}$. Effector CD8 ^+^ T cells have a finite lifespan and die within the tumor microenvironment as a rate equal to *k*_*d*5_·*T*_*E*3_. The influx rate of effector CD8 ^+^*T* cells from the blood to tumor is defined by $a_{23}\frac {T_{E2}Vol_{b}}{Vol_{t}}$, where *Vol*_*t*_=*ε*+*s*_*t*_*C*(*t*)+*V*_*i*_*T*_*E*3_*mm*^3^ is the volume of the tumor compartment, *ε* is a small positive constant representing a small volume of tissue that excludes tumor and effector CD8 ^+^ T cells in the tumor compartment, *s*_*t*_=6∗10^−7^*mm*^3^ is the average size of a B16F10 tumor cell ([41]), and *V*_*i*_=10^−7^*mm*^3^ is the average size of a T effector cell ([42]). The efflux rate of effector CD8 ^+^ T cells from the tumor to blood is $a_{32}T_{E3}\frac {C_{MHCI^{-}}}{\epsilon +C(t)}$.
- **Interferon gamma (IFN*γ*, units: moles per mm**^**3**^**)**. We assume that Interferon *γ* is secreted solely by effector CD8 ^+^ T cells within the tumor at a rate proportional to the concentration of effector CD8 ^+^ T cells within the tumor microenvironment and decays at a rate proportional to its concentration. While this assumption may not hold in all model systems, the presence of IFN *γ* in the tumor was dependent on CD8 ^+^ T cell activation [43].
- **Tumor Necrosis Factor*α* (TNF*α*, units: moles per mm**^**3**^**)**. We assume that Tumor Necrosis Factor *α* decays naturally at a rate proportional to its concentration and is secreted solely by effector CD8 ^+^ T cells in the tumor at a rate that includes both autocrine and constitutive production terms: $\left (k_{c2}\frac {\text {TNF}{\alpha }}{k_{2}+\text {TNF}{\alpha }}+k_{3}\right)T_{E3}$. While this assumption may not hold in all model systems, the presence of TNF *α* in the tumor was also dependent on CD8 ^+^ T cell activation [43].

Our mathematical model is based on the experimental data presented by Bramson and coworkers [15, 16] using the B16F10 model for metastatic melanoma. The B16F10 model is one of a number of transplantable models of cancer that have been used as pre-clinical models to test anti-tumor immunotherapies [35]. In these models, a malignant cell line derived from a spontaneous mouse cancer is cultured in vitro and then injected back into syngeneic immunocompetent mice. The B16 model was used to help demonstrate the efficacy of anti-CTLA4 therapy [36], a drug that has revolutionized the treatment of metastatic melanoma in humans [4, 37]. As an alternative approach to enhance anti-tumor immunity, Bramson and coworkers examined how a cytotoxic CD8 ^+^ T cell response directed against a tumor antigen using a recombinant adenovirus vector can help control tumor growth. This adenovirus vector induces both the transient expression of a defined tumor antigen and triggers innate immunity to initiate a primary adaptive immune response against this tumor antigen. A primary adaptive immune response is organized spatially: presentation of antigen and initial activation of naïve CD8 ^+^ T cells occurs in the secondary lymphoid organs, activated effector T cells circulate in the blood and peripheral tissues in search of tumor antigens, and effector T cells remain in tissues that express tumor antigens and selectively kill cells that express the cognate tumor antigen [38]. In order to better understand the dynamics of the primary response to adenovirus-mediated induction of an anti-tumor immune response, we developed a three-compartment mathematical mode to quantify the cytotoxic CD8 ^+^ T cell response to adenovirus vaccination and subsequent inhibition of tumor cell growth, as shown schematically in Fig. 1. Among these three compartments, we consider the dynamics of nine state variables that are regulated by the following governing biological processes and assumptions:

- **Naïve CD8**^**+**^** T cells (*T***_***N***_**, units: cells per mm**^**3**^**)**. As the immunization protocol induces the clonal expansion of small subset of CD8 ^+^ T cell clones rather than globally changing T cell numbers, we assumed that naïve CD8 ^+^ T cells expressing the T cell receptor that recognizes the epitope derived from the immunized tumor antigen are produced at a constant rate *c*_1_ from thymus and die naturally at a rate *k*_*d*1_*T*_*N*_. Naïve CD8 ^+^ T cells are maintained at a constant level in the absence of adenovirus, i.e., *c*_1_=*k*_*d*1_*T*_*N*_(0). Naïve CD8 ^+^ T cells are recruited to the lymph node and activated by adenovirus vaccination and become effector CD8 ^+^ T cells (*T*_*E*1_) when they encounter adenovirus-induced antigen expression (LV) at a rate proportional to *T*_*N*_ and a saturable adenovirus-induced antigen (LV) term defined by $\frac {\text {LV}}{\text {LV}+\gamma }$.

Based on the governing biological processes and assumptions described above, the dynamics of these cytotoxic T cell, tumor cell, and cytokine state variables are represented by a mass-action formalism and encoded by the following impulsive ordinary differential equations:

(1) $$ \frac{dT_{N}}{dt}=c_{1}-k_{d1} T_{N}-c_{2}T_{N} \frac{\text{LV}}{\text{LV}+\gamma},  $$

(2) $$\begin{array}{*{20}l} \frac{dT_{E1}}{dt}&=c_{2} \frac{T_{N}Vol_{b}}{Vol_{ln}}\frac{\text{LV}}{\text{LV}+\gamma}+ k_{p1}T_{E1} \frac{\text{LV}}{\text{LV}+\gamma}\frac{\alpha}{\alpha+T^{2}_{E1}}\\&\quad+a_{21}\frac{T_{E2}Vol_{b}}{Vol_{ln}}-a_{12}T_{E1}, \end{array} $$

(3) $$ \begin{aligned} \frac{d\text{LV}}{dt}&=-k_{d2}\text{LV},\quad t\neq t_{k}, \;k=1,2,\ldots,n,\\ \end{aligned}  $$

(4) $$ \begin{aligned} \frac{dT_{E2}}{dt}&=-k_{d3} T_{E2} +a_{12}\frac{T_{E1}Vol_{ln}}{Vol_{b}} -a_{21}T_{E2}\\&\quad+a_{32}\frac{C_{MHCI^{-}}}{\epsilon+C(t)}\frac{T_{E3}Vol_{t}}{Vol_{b}}-a_{23}T_{E2},\\ \end{aligned}  $$

(5) $$\begin{array}{*{20}l} \frac{dC_{MHCI^{+}}}{dt}&=c_{3}\frac{\text{IFN}_{\gamma}}{k_{1} +\text{IFN}_{\gamma}}C_{MHCI^{-}}-k_{p2} C_{MHCI^{+}}\\ &\qquad-k_{d4} C_{MHCI^{+}} - c_{4} T_{E3}\frac{C_{MHCI^{+}}}{\epsilon+C(t)}, \end{array} $$

(6) $$\begin{array}{*{20}l} \frac{dC_{MHCI^{-}}}{dt}&=-c_{3} \frac{\text{IFN}_{\gamma}}{k_{1} +\text{IFN}_{\gamma}}C_{MHCI^{-}}-k_{d4} C_{MHCI^{-}}\\ &\qquad+k_{p2} C_{MHCI^{-}}-r_{2} C^{2}_{MHCI^{-}}\\ &\qquad+2k_{p2} C_{MHCI^{+}}, \end{array} $$

(7) $$\begin{array}{*{20}l} \frac{dT_{E3}}{dt}&=a_{23}\frac{T_{E2}Vol_{b}}{Vol_{t}}-a_{32}T_{E3}\frac{C_{MHCI^{-}}}{\epsilon+C(t)}\\ &\qquad+ k_{p3}\frac{C_{MHCI^{+}}}{\epsilon+C(t)}T_{E3}-k_{d5} T_{E3}, \end{array} $$

(8) $$ \begin{aligned} \frac{d \text{IFN}_{\gamma}}{dt}=-k_{d6}\text{IFN}_{\gamma}+k_{c1}T_{E3},\\ \end{aligned}  $$

(9) $$ \begin{aligned} \frac{d \text{TNF}_{\alpha}}{dt}=-k_{d7} \text{TNF}_{\alpha}+k_{c2}\frac{\text{TNF}_{\alpha}}{k_{2}+\text{TNF}_{\alpha}}T_{E3}+k_{3}T_{E3}, \end{aligned}  $$

(10) $$ \begin{aligned} \Delta \text{LV}(t)=\text{LV}_{k}, \;\;\;t=t_{k},\;\;k=1,2, 3,\cdots, n, \end{aligned}  $$

where *Δ*LV(*t*)=LV(*t*^+^)−LV(*t*^−^) reflects the abrupt change of adenovirus at vaccination time t and LV_*k*_ is the dosage of the adenovirus vaccination at the administration time *t*_*k*_ with *k*=1,2,3,⋯,*n*.

### Model calibration

Next, we calibrated model parameters against self-consistent experimental data. These data were acquired from two papers. The first paper described the general dynamics of a CD8 ^+^ T cell response to vaccination with a recombinant human adenovirus serotype 5 (rHuAd5) vector that can be used as a general delivery vehicle to express human tumor antigens [16]. The second paper describes using this adenovirus vector to induce a CD8 ^+^ T cell response to the human dopachrome tautomerase antigen (hDCT; vector: rHuAd5-hDCT) [15]. In contrast, the same adenovirus vector engineered to vaccinate against the glycoprotein gp100 (rHuAd5-hgp100) was unable to control the growth of B16F10 in prophylactic and neo-adjuvant settings. The B16F10 cell line exhibits a defect in the processing and presentation of peptides derived from gp100 through the Major Histocompatibility Complex class I pathway [44]. Together these results suggest that the control of tumor growth induced by rHuAd5-hDCT is through tumor-specific CD8 ^+^ T cells.

The experimental data used in calibrating the mathematical model are listed as follows:

- CD8 ^+^ T cells in the secondary lymph nodes (*T*_*E*1_) and effector CD8 ^+^ T cells in the blood (*T*_*E*2_) are obtained from Figure 1(A) of Yang’s paper ([16]).
- Antigen expression derived from adenovirus vaccination (LV) corresponds to data presented in Figure 3(B) of Yang’s paper ([16]).
- Total volume of B16F10-derived tumors was calibrated against data shown in Figure 1(B) of McGray’s paper ([15]).
- The concentration of effector CD8 ^+^ T cells present within the tumor (*T*_*E*3_) are found in Figure 4(A) of McGray’s paper ([15]).
- Expression of Interferon gamma ($\overline {\text {IFN}} \gamma $) and Tumor Necrosis Factor alpha ($\overline {\text {TNF}}\alpha $) genes within the tumor are obtained from Figure 1(E) of McGray’s paper ([15]).

As there are more data points (93) than parameters (27) parameters, the mechanistic model is identifiable in theory.

**Fig. 2:**
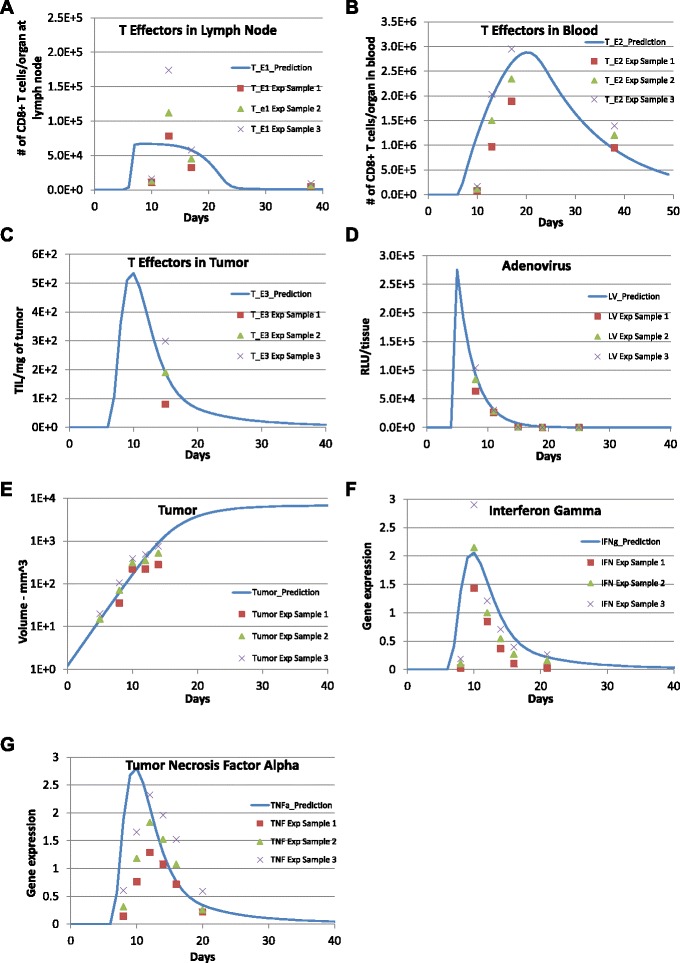
Model predictions are compared with experimental data. Comparison between model predictions (solid lines) and experimental data (symbols) reported for (**a**) CD8+ T cells lymph nodes (Fig 1A in [16]), (**b**) CD8+ T cells in blood (Fig 1A in [16]), (**c**) CD8+ T cells in tumor (Fig 4A in [15]), (**d**) antigen expression derived adenovirus vector (Fig 3B in [16]), (**e**) B16 tumor volume (Fig 1B in [15]), (**f**) Interferon-gamma gene expression in tumor (Fig 1E in [15]), (**g**) Tumor necrosis factor alpha gene expression in tumor (Fig 1E in [15]). The symbols represent the mean +/- SEM. Values of calibrated model parameters can be found in Table 1

Simulation results for the modeled variables along with their corresponding experimental measurements are shown in Fig. 2, where *t*_0_=0 is the day of tumor inoculation, *t*=5 is the day of adenovirus immunization. The starting concentration of naïve CD8 ^+^ T cells ($T_{N}(0)=0.0714 \,\text {cells}\,\text {per}\,\text {mm}^{3}$) was estimated by assuming that the number of naïve CD8 ^+^ T cells in a mouse is 100 and the volume of the blood system of a mature mouse is 1.4∗10^3^*mm*^3^). Initially, 2×10^6^ tumor cells were injected into mice and assumed to not express tumor antigens ($C_{MHCI^{-}}\phantom {\dot {i}\!}$). The remaining state variables were initially zero. Vaccination was simulated by abruptly changing the concentration of adenovirus (*LV*) at the administration time (*t*_1_=5, the 5th day after tumor implantation) using an impulse dose equal to LV_1_ ($\text {LV}_{1}=1.100\times 10^{6} \;\text {RLU}\,\text {per}\, \text {mm}^{3}$). The calibrated parameter values obtained using the genetic algorithm are listed in Table 1.

**Table 1:** Parameter values determined by calibrating model against experimental data

Parameter	Units	Description	Calibrated values
*k*_*d*1_	*d* *a* *y* ^−1^	Naïve CD8 ^+^ T cell natural death rate constant	3.809×10^−3^
*k*_*d*2_	*d* *a* *y* ^−1^	Adenovirus natural death rate constant	0.364
*k*_*d*3_	*d* *a* *y* ^−1^	Blood T effector natural death rate constant	1.80×10^−2^
*k*_*d*4_	*d* *a* *y* ^−1^	Tumor cell natural death rate constant	2.08×10^−6^
*k*_*d*5_	*d* *a* *y* ^−1^	Tumor T effector natural death rate constant	0.800
*k*_*d*6_	*d* *a* *y* ^−1^	Interferon *γ* natural degradation rate constant	0.082
*k*_*d*7_	*d* *a* *y* ^−1^	Tumor Necrosis Factor *α* natural degradation rate constant	3.10×10^−6^
*k*_*p*1_	*d* *a* *y* ^−1^	Lymph node T effector proliferation rate constant due to adenovirus vaccination	12.017
*k*_*p*2_	*d* *a* *y* ^−1^	Tumor cell proliferation rate constant	0.5
*k*_*p*3_	*d* *a* *y* ^−1^	Tumor T effector proliferation rate constant due to tumor growth	5.73×10^−6^
*a*_12_	*d* *a* *y* ^−1^	Rate constant for T cell flow from lymph node to blood	5.706
*a*_21_	*d* *a* *y* ^−1^	Rate constant for T cell flow from blood to lymph node	3.540×10^−3^
*a*_23_	*d* *a* *y* ^−1^	Rate constant for T cell flow from blood to tumor	5.546×10^−2^
*a*_32_	*d* *a* *y* ^−1^	Rate constant for T cell flow from tumor to blood	6.89×10^−18^
*c*_1_	*c* *e* *l* *l*·*m* *m* ^−3^·*d* *a* *y* ^−1^	Naïve T cell natural production rate	2.719×10^−4^
*c*_2_	*d* *a* *y* ^−1^	T cell to lymph node T effector transfer rate constant	0.5263
*c*_3_	*d* *a* *y* ^−1^	MHC class I negative to positive tumor cells transfer rate constant	0.8759
*c*_4_	*m* *m* ^3^·*d* *a* *y* ^−1^	MHCI positive tumor death rate due to T effector (in tumor) lysis	2.49×10^−13^
*α*	(*c* *e* *l* *l*·*m* *m* ^−3^)^2^	Lymph node T effector saturation constant	6.520×10^10^
*k*_1_	*m* *o* *l* *e* *s*·*m* *m* ^−3^	Interferon *γ* saturation constant	3.69×10^−9^
*k*_2_	*m* *o* *l* *e* *s*·*m* *m* ^−3^	Tumor Necrosis Factor *α* saturation constant	6.924×10^6^
*k*_3_	*m* *o* *l* *e* *s*·*d* *a* *y* ^−1^·*c* *e* *l* *l* ^−1^	Constitutive Tumor Necrosis Factor *α* production rate constant	2.634×10^−4^
*k*_*c*1_	*m* *o* *l* *e* *s*·*d* *a* *y* ^−1^·*c* *e* *l* *l* ^−1^	Cellular Interferon *γ* production rate constant	7.295×10^8^
*k*_*c*2_	*m* *o* *l* *e* *s*·*d* *a* *y* ^−1^·*c* *e* *l* *l* ^−1^	Autocrine Tumor Necrosis Factor *α* production rate constant	9.939×10^8^
*γ*	*R* *L* *U*·*m* *m* ^−3^	Adenovirus saturation constant	2.905×10^3^
*β*_1_	AU	Constant of proportionality in calculating *T* *C* *R*_*α*_ gene expression	8.79×10^−6^
*r*_2_	*c* *e* *l* *l* ^−1^·*d* *a* *y* ^−1^	Constant in tumor logistic growth (or MHC class I negative tumor growth rate divided by the carrying capacity *K*, i.e., $\frac {k_{p2}-k_{d4}}{K}$)	3.34×10^−10^

### Stability analysis

The dynamics of the nonlinear ODE model comprised of equations (1) - (9) are complicated. To gain insight into the behavior of the system, we explored the steady state solutions of the system using stability analysis. By setting the right hand sides of the ODE system (1) - (9) to 0 and solving the equations simultaneously, we notice that the system of ODEs (1) - (9) only has a tumor-free equilibrium $\overrightarrow {X}_{0}$:

$$\begin{array}{@{}rcl@{}} \overrightarrow{X}_{0}&=&\left(T_{N},\;T_{E1},\;LV,\,T_{E2},\;C_{MHCI}^{+}, \;C_{MHCI}^{-},\; T_{E3},\right.\\ &&\left.\text{IFN}_{\gamma}, \;\text{TNF}_{\alpha}\right)^{T}\\ &=&\left(\frac{c_{1}}{k_{d1}},0,0,0,0,0,0,0,0\right)^{T}, \end{array} $$

and a high-tumor equilibrium $\overrightarrow {X}_{1}$:

$$\begin{array}{@{}rcl@{}} \overrightarrow{X}_{1}&=&\left(T_{N},\;T_{E1},\;LV,\,T_{E2},\;C_{MHCI}^{+}, \;C_{MHCI}^{-},\; T_{E3},\right.\\ &&\left.\text{IFN}_{\gamma}, \;\text{TNF}_{\alpha}\right)^{T}\\ &=&\left(\frac{c_{1}}{k_{d1}},0,0,0,0,\frac{k_{p2}-k_{d4}}{r_{2}},0,0,0\right)^{T}. \end{array} $$

We note that the tumor-free equilibrium has only one none-zero element: the naïve T cells *T*_*N*_, this occurs when there are no tumor cells present and no adenovirus immunization treatment is administered and also corresponds to tumor-specific effector CD8 ^+^ T cells and cytokines being equal to zero. The high tumor equilibrium $\overrightarrow {X}_{1}$ has two non-zero elements: the naïve T cells *T*_*N*_ and the MHC class I negative tumor cells $\phantom {\dot {i}\!}C_{\textit {MHCI}}^{-}$, which reflects the status of the steady state when the one-time adenovirus vaccination treatment failed to completely eradicate the tumor cells. This situation occurs when adenovirus *LV* decays to zero and the MHC class I positive tumor cells are all killed by tumor infiltrating lymphocytes, which causes exhaustion of effector CD8 ^+^ T cells in three compartments and cytokines decay to zero. The rest of the MHC class I negative tumor cells then approach the carrying capacity and the naïve T cells return to their original constant level.

By simple calculation, we obtain the Jacobian matrix $J(\overrightarrow {X})$ of the ODE system (1)- (9):

$${\fontsize{8.5}{6} \left[\begin{array}{ccccccccc} J_{11} & 0 &-\frac{c_{2}\gamma T_{N}}{(LV+\gamma)^{2}} & 0 & 0 & 0 & 0 & 0 & 0 \\[-1pt] J_{21} & J_{22} & J_{23} & \frac{a_{21}Vol_{b}}{Vol_{ln}} & 0 & 0 & 0 & 0 & 0\\[-1pt] 0 & 0 & -k_{d2} & 0 & 0 & 0 & 0 & 0 & 0 \\[-1pt] 0 & \frac{a_{12}Vol_{ln}}{Vol_{b}} & 0 & J_{44} & J_{45} & J_{46} & J_{47} & 0 & 0 \\[-1pt] 0 & 0 & 0 & 0 & J_{55} & J_{56} & J_{57} &\frac{k_{1}c_{3}C_{MHCI}^{-}}{(k_{1}+\text{IFN}_{\gamma})^{2}}&0\\ 0 & 0 & 0 & 0 & 2k_{p2} & J_{66} & 0 &-\frac{k_{1}c_{3}C_{MHCI}^{-}}{(k_{1}+\text{IFN}_{\gamma})^{2}} & 0\\[-1pt] 0 & 0 & 0 & J_{74} & J_{75} & J_{76} & J_{77} & 0 & 0 \\[-1pt] 0 & 0 & 0 & 0 & 0 & 0 & k_{c1} & -k_{d6} & 0 \\[-1pt] 0 & 0 & 0 & 0 & 0 & 0 & J_{97} & 0 & J_{99} \end{array} \right],} $$

where

$$\begin{array}{@{}rcl@{}}\vspace*{-15pt} \overrightarrow{X}&=& \left(T_{N},\;T_{E1},\;\text{LV},\,T_{E2},\;C_{MHCI}^{+}, \;C_{MHCI}^{-},\; T_{E3},\;\text{IFN}_{\gamma}, \;\text{TNF}_{\alpha}\right)^{T},\\[-1pt] J_{11}&=&-k_{d1}-c_{2}\frac{\text{LV}}{\text{LV}+\gamma},\\[-1pt] J_{21}&=&\frac{c_{2}Vol_{b} LV}{Vol_{ln}(LV+\gamma)},\\ [-1pt] J_{22}&=&\frac{k_{p1}LV}{LV+\gamma}\left(\frac{\alpha}{\alpha+T^{2}_{E1}}\right)+k_{p1}T_{E1}\frac{\text{LV}}{\text{LV}+\gamma}\left(\frac{-2\alpha T_{E1}}{\left(\alpha+T_{E1}^{2}\right)^{2}}\right)-a_{12},\\[-1pt] J_{23}&=&\frac{c_{2}T_{N}\gamma Vol_{b}}{Vol_{ln}\left(\text{LV}+\gamma\right)^{2}}+\frac{k_{p1}\gamma T_{E1}}{\left(\text{LV}+\gamma\right)^{2}}\left(\frac{\alpha}{\alpha+T_{E1}^{2}}\right),\\[-1pt] J_{44}&=&-k_{d3}-a_{21}-a_{23},\\[-1pt] J_{45}&=&\frac{a_{32}C_{MHCI}^{-} T_{E3}\left(\epsilon\left(s_{t}-1\right)-V_{i}T_{E3}\right)}{Vol_{b}\left(\epsilon+C_{MHCI}^{+}+C_{MHCI}^{-}\right)^{2}},\\ J_{46}&=&\frac{a_{32}T_{E3}\left[\left(\epsilon+C_{MHCI}^{+}\right)(\epsilon+s_{t}C_{MHCI}^{+}+2s_{t}C_{MHCI}^{-}+V_{i}T_{E3})+s_{t}(C_{MHCI}^{-})^{2}\right]}{Vol_{b}\left(\epsilon+C_{MHCI}^{+}+C_{MHCI}^{-}\right)^{2}},\\ J_{47}&=&\frac{a_{32}C_{MHCI}^{-}\left(\epsilon+s_{t}C_{MHCI}^{+}+s_{t}C_{MHCI}^{-}+2V_{i}T_{E3}\right)}{\left(\epsilon+C_{MHCI}^{+}+C_{MHCI}^{-}\right)Vol_{b}},\\ J_{55}&=&-k_{p2}-k_{d4}-c_{4}T_{E3}\frac{\epsilon+C_{MHCI}^{-}}{\left(\epsilon+C_{MHCI}^{+}+C_{MHCI}^{-}\right)^{2}},\\ J_{56}&=&\frac{c_{3}\text{IFN}_{\gamma}}{k_{1}+\text{INF}_{\gamma}}+\frac{c_{4}T_{E3}C_{MHCI}^{+}}{\left(\epsilon+C_{MHCI}^{+}+C_{MHCI}^{-}\right)^{2}},\\ J_{57}&=&-\frac{c_{4}C_{MHCI}^{+}}{\epsilon+C_{MHCI}^{+}+C_{MHCI}^{-}},\\ J_{66}&=&-c_{3}\frac{\text{IFN}_{\gamma}}{\text{IFN}_{\gamma}+k_{1}}-k_{d4}-2r_{2}C_{MHCI}^{-}+k_{p2},\\ J_{74}&=&\frac{a_{23}Vol_{b}}{\epsilon+s_{t}C_{MHCI}^{+}+s_{t}C_{MHCI}^{-}+V_{i}T_{E3}},\\ J_{75}&=&-\frac{a_{23}s_{t}T_{E2}Vol_{b}}{\left(\epsilon+s_{t}C_{MHCI}^{+}+s_{t}C_{MHCI}^{-}+V_{i}T_{E3}\right)^{2}}+\frac{a_{32}T_{E3}C_{MHCI}^{-}}{\left(\epsilon+C_{MHCI}^{+}+C_{MHCI}^{-}\right)^{2}}\\ &&+\frac{k_{p3}T_{E3}\left(\epsilon+C_{MHCI}^{-}\right)}{\left(\epsilon+C_{MHCI}^{+}+C_{MHCI}^{-}\right)^{2}},\\ J_{76}&=&-\frac{a_{23}s_{t}T_{E2}Vol_{b}}{\left(\epsilon+s_{t}C_{MHCI}^{+}+s_{t}C_{MHCI}^{-}+V_{i}T_{E3}\right)^{2}}-\frac{a_{32}T_{E3}\left(\epsilon+C_{MHCI}^{+}\right)}{\left(\epsilon+C_{MHCI}^{+}+C_{MHCI}^{-}\right)^{2}}\\ &&-\frac{k_{p3}T_{E3}C_{MHCI}^{+}}{\left(\epsilon+C_{MHCI}^{+}+C_{MHCI}^{-}\right)^{2}},\\ J_{77}&=&-\frac{a_{23}V_{i} T_{E2}Vol_{b}}{\left(\epsilon+s_{t}C_{MHCI}^{+}+s_{t}C_{MHCI}^{-}+V_{i}T_{E3}\right)^{2}}-k_{d5}-\frac{a_{32}C_{MHCI}^{-}}{\epsilon+C_{MHCI}^{+}+C_{MHCI}^{-}}\\ &&+\frac{k_{p3}C_{MHCI}^{+}}{\epsilon+C_{MHCI}^{+}+C_{MHCI}^{-}},\\ J_{97}&=&\frac{k_{c2}\text{TNF}_{\alpha}}{k_{2}+\text{TNF}_{\alpha}}+k_{3},\\ J_{99}&=&-k_{d7}+\frac{k_{c2}k_{2}T_{E3}}{\left(k_{2}+\text{TNF}_{\alpha}\right)^{2}}. \end{array} $$

The Jacobian matrix evaluated at the tumor-free equilibrium $J(\overrightarrow {X}_{0})$ is given by

$${\fontsize{7.5}{6} \left[ \begin{array}{ccccccccc} -k_{d1} & 0 &-\frac{c_{1}c_{2}}{\gamma k_{d1}} & 0 & 0 & 0 & 0 & 0 & 0 \\ 0 & -a_{12} & \frac{c_{1}c_{2}Vol_{b}}{\gamma k_{d1}Vol_{ln}} & \frac{a_{21}Vol_{b}}{Vol_{ln}} & 0 & 0 & 0& 0 & 0 \\ 0 & 0&-k_{d2} & 0 & 0 & 0 & 0 & 0 & 0 \\ 0 & \frac{a_{12}Vol_{ln}}{Vol_{b}} & 0 & J_{44} & 0 & 0 & 0 & 0 & 0 \\ 0 & 0 & 0 & 0 & J_{55} & 0 & 0 & 0 & 0 \\ 0 & 0 & 0 & 0 & 2k_{p2} & k_{p2}-k_{d4} & 0 & 0 & 0 \\ 0 & 0 & 0 & \frac{a_{23}Vol_{b}}{\epsilon} & 0 & 0 & -k_{d5} & 0 & 0 \\ 0 & 0 & 0 & 0 & 0 & 0 & k_{c1} & -k_{d6} & 0 \\ 0 & 0 & 0 & 0 & 0 & 0 & k_{3} & 0 & -k_{d7} \end{array} \right],} $$

where *J*_44_=−*k*_*d*3_−*a*_21_−*a*_23_ and *J*_55_=−*k*_*p*2_−*k*_*d*4_.

It can be shown that $J(\overrightarrow {X}_{0})$ has the following eigenvalues: *λ*_1_=−*k*_*d*1_, *λ*_2_=−*k*_*d*2_, *λ*_3_=−*k*_*d*5_, *λ*_4_=−*k*_*d*6_, *λ*_5_=−*k*_*d*7_, *λ*_6_=−*k*_*p*2_−*k*_*d*4_, *λ*_7_=*k*_*p*2_−*k*_*d*4_, while *λ*_8_ and *λ*_9_ are determined by the quadratic equation

(11) $$\begin{array}{*{20}l} \lambda^{2}+(a_{12}+a_{21}+a_{23}+k_{d3})\lambda+a_{12}(k_{d3}+a_{23})=0. \end{array} $$

Let *Δ*=(*a*_12_+*a*_21_+*a*_23_+*k*_*d*3_)^2^−4*a*_12_(*k*_*d*3_+*a*_23_). Then if *Δ*≥0, equation (11) has the real roots

$$\begin{aligned} &\lambda_{8}=\frac{-(a_{12}+a_{21}+a_{23}+k_{d3})+\sqrt{\Delta}}{2},\;\;\text{and}\\ &\lambda_{8}=\frac{-(a_{12}+a_{21}+a_{23}+k_{d3})-\sqrt{\Delta}}{2}; \end{aligned} $$

if *Δ*<0, equation (11) has the complex conjugate roots

$$\begin{aligned} &\lambda_{8}=\frac{-(a_{12}+a_{21}+a_{23}+k_{d3})+i\sqrt{-\Delta}}{2},\;\;\text{and}\\ &\lambda_{8}=\frac{-(a_{12}+a_{21}+a_{23}+k_{d3})-i\sqrt{-\Delta}}{2}. \end{aligned} $$

In both cases, *λ*_8_ and *λ*_9_ have negative real parts.

Thus when *k*_*p*2_>*k*_*d*4_, the tumor-free equilibrium $\overrightarrow {X}_{0}$ is unstable and when *k*_*p*2_<*k*_*d*4_, the tumor-free equilibrium $\overrightarrow {X}_{0}$ is stable since all eigenvalues of the Jacobian matrix have negative real parts.

The Jacobian matrix evaluated at the high tumor equilibrium $J(\overrightarrow {X}_{1})$ is given by

$${\fontsize{7.8}{6} \left[ \begin{array}{ccccccccc} -k_{d1} & 0 &-\frac{c_{1}c_{2}}{\gamma k_{d1}} & 0 & 0 & 0 & 0 & 0 & 0 \\ 0& -a_{12} & \frac{c_{1}c_{2}Vol_{b}}{\gamma k_{d1}Vol_{ln}}&\frac{a_{21}Vol_{b}}{Vol_{ln}}&0&0&0&0&0 \\ 0& 0&-k_{d2} & 0 & 0 & 0 & 0 & 0 & 0 \\ 0& \frac{a_{12}Vol_{ln}}{Vol_{b}}&0&J_{44}&0&0&J_{47}&0&0 \\ 0& 0&0&0&J_{55}&0&0&J_{58}&0 \\ 0& 0&0&0&2k_{p2}&J_{66}&0&J_{68}&0 \\ 0& 0&0&\frac{a_{23}r_{2}Vol_{b}}{r_{2} \epsilon+s_{t}(k_{p2}-k_{d4})}&0&0&J_{77}&0&0 \\ 0& 0&0&0&0&0&k_{c1}&-k_{d6}&0 \\ 0& 0&0&0&0&0&k_{3}&0&-k_{d7} \end{array} \right],} $$

where

$$\begin{array}{@{}rcl@{}} J_{44}&=& -k_{d3}-a_{21}-a_{23},\\ J_{47}&=&\frac{a_{32}(k_{p2}-k_{d4})(r_{2}\epsilon+s_{t}(k_{p2}-k_{d4}))}{(r_{2}\epsilon+s_{t}(k_{p2}-k_{d4}))Vol_{b}},\\ J_{55}&=& -k_{p2}-k_{d4},\\ J_{58}&=&\frac{c_{3}(k_{p2}-k_{d4})}{r_{2}k_{1}},\\ J_{66}&=& -(k_{p2}-k_{d4}),\\ J_{68}&=&-\frac{c_{3}(k_{p2}-k_{d4})}{r_{2}k_{1}},\\ J_{77}&=&-k_{d5}-\frac{a_{32}(k_{p2}-k_{d4})}{r_{2}\epsilon+k_{p2}-k_{d4}}. \end{array} $$

It can be shown that $J(\overrightarrow {X}_{1})$ has the following eigenvalues: *λ*_1_=−*k*_*d*1_, *λ*_2_=−*k*_*d*2_, *λ*_3_=−*k*_*d*6_, *λ*_4_=−*k*_*d*7_, *λ*_5_=−*k*_*p*2_−*k*_*d*4_, *λ*_6_=−(*k*_*p*2_−*k*_*d*4_), and *λ*_7_, *λ*_8_ and *λ*_9_ are determined by

(12) $$\begin{array}{@{}rcl@{}} &&(\lambda+a_{12})\{(\lambda+k_{d3}+a_{21}+a_{23})[(\lambda+k_{d5})(r_{2}\epsilon+k_{p2}\\&&-k_{d4})+a_{32}(k_{p2}-k_{d4})]\\ &&-a_{23}a_{32}(k_{p2}-k_{d4})\}=a_{12}a_{21}[(\lambda+k_{d5})(r_{2}\epsilon+k_{p2}\\&&-k_{d4})+a_{32}(k_{p2}-k_{d4})]. \\ &&\end{array} $$

Since $\epsilon \doteq 0$, equation (12) becomes the cubic equation

(13) $$\begin{array}{@{}rcl@{}} \lambda^{3}+a_{2}\lambda^{2}+a_{1}\lambda+a_{0} = 0, \end{array} $$

where *a*_2_=*a*_12_+*a*_21_+*a*_23_+*a*_32_+*k*_*d*3_+*k*_*d*5_, *a*_1_=*a*_12_(*k*_*d*5_+*a*_32_+*k*_*d*3_+*a*_23_)+*k*_*d*3_(*k*_*d*5_+*a*_32_)+*a*_21_(*k*_*d*5_+*a*_32_)+*k*_*d*5_*a*_23_, and *a*_0_=*a*_12_(*k*_*d*3_*k*_*d*5_+*a*_32_*k*_*d*3_+*a*_23_*k*_*d*5_). It is easy to see that *a*_*i*_>0 for *i*=0,1,2 since all parameters are positive. By simple calculation, we can obtain *a*_2_*a*_1_−*a*_0_>0 which implies that roots of equation (13) (*λ*_7_, *λ*_8_, *λ*_9_) all have negative real parts by the Routh-Hurwitz criterion. Thus when the proliferation rate of tumor cells is greater than the natural death rate of tumor cells (i.e., *k*_*p*2_>*k*_*d*4_), the high tumor equilibrium $\overrightarrow {X}_{1}$ is stable and when the proliferation rate of tumor cells is less than the natural death rate of tumor cells (i.e., *k*_*p*2_<*k*_*d*4_), the high-tumor equilibrium $\overrightarrow {X}_{1}$ is unstable.

Therefore, using the parameter values obtained from model calibration (*k*_*p*2_>*k*_*d*4_), the tumor-free equilibrium $\overrightarrow {X}_{0}$ is unstable and the high tumor equilibrium $\overrightarrow {X}_{1}$ is stable. This implies that under the current status of the mouse immune system, a small tumor will keep growing to its carrying capacity because of the fast proliferation of tumor cells without adenovirus vaccination treatment. On the other hand, the one-time adenovirus immunization as applied in the experiment was not very successful in completely eliminating tumor cells due to limited effects on enhancing CTL immune response. It seems that the CTL response falls to zero before all MHC negative tumor cells are converted to MHC positive tumor cells and killed by the cytotoxic CD8 ^+^ T cells. Then, MHC positive tumor cells approach zero while MHC negative tumor cells approach the carrying capacity and all T cell effectors and cytokines drop back to zero soon after the one time vaccination treatment (see Fig. 2).

### Effect of multiple vaccinations

Next, we investigated in silico the impact of multiple adenovirus vaccinations on T cell proliferation and recruitment, cytokine secretion, and tumor growth using the calibrated model in conjunction with an impulsive control mechanism where control laws are discrete in time, as represented by Eq. (10).

**Fig. 3:**
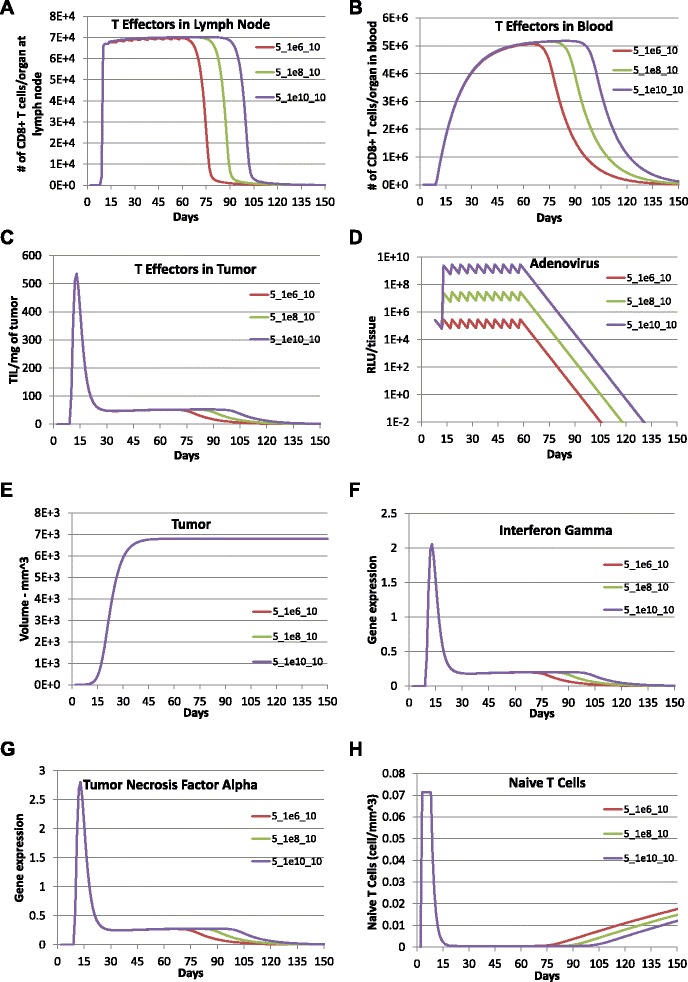
Prediction of effect of multiple vaccinations with increasing dosage using the calibrated model. Model predictions were obtained with adenovirus vaccination with a dose of 1×10^10^ RLU per mm^3^ (purple lines), a dose of 1×10^8^ RLU per mm^3^ (green lines), and a dose of 1×10^6^ RLU per mm^3^ (red lines) every 5 days for a total of 10 times following the initial vaccination with a dose of 1.1×10^6^ RLU per mm^3^ on day 5 after tumor implantation. **a** TE1, **b** TE2, **c** TE3, **d** LV, **e** Total tumor cells (CMHCI+ + CMHCI-), **f** IFNG, **g** TNFA, and **h** TN

To explore the impact of dose-dependence, adenovirus vaccinations were simulated with an increasing dosage of 1×10^6^ RLU per mm^3^, 1×10^8^ RLU per mm^3^, and 1×10^10^ RLU per mm^3^ and delivered every 5 days for a total of 10 times following the initial vaccination (see Fig. 3). The simulation results indicate that with the same frequency and number of times of immunizations, the increase in immunization dose increases the length and the maximum magnitude of the adenovirus vaccinations and CTL response. As we can see in Fig. 3, a 100-fold increase in immunization dose results in a 15-day (or 10 %) increase in the length of the adenovirus vaccinations as well as CTL response. However, the impact of increase in vaccination dose on the strength of CTL response is very limited: a 100-fold increase in immunization dose generates less than a 1 % increase in the collective magnitude of T cell concentration in all three compartments. The increased immunization dosage shows a significant impact on the length but not the collective magnitude of Interferon gamma (IFNG) and Tumor Necrosis Factor alpha (TNF *α*) in the tumor microenvironment. Surprisingly, the increase in dose with multiple immunizations does not appear to affect the peak time and maximum magnitude of tumor growth. In addition, the time period of tumor staying at its carrying capacity barely changes with increased dose of vaccinations.

**Fig. 4:**
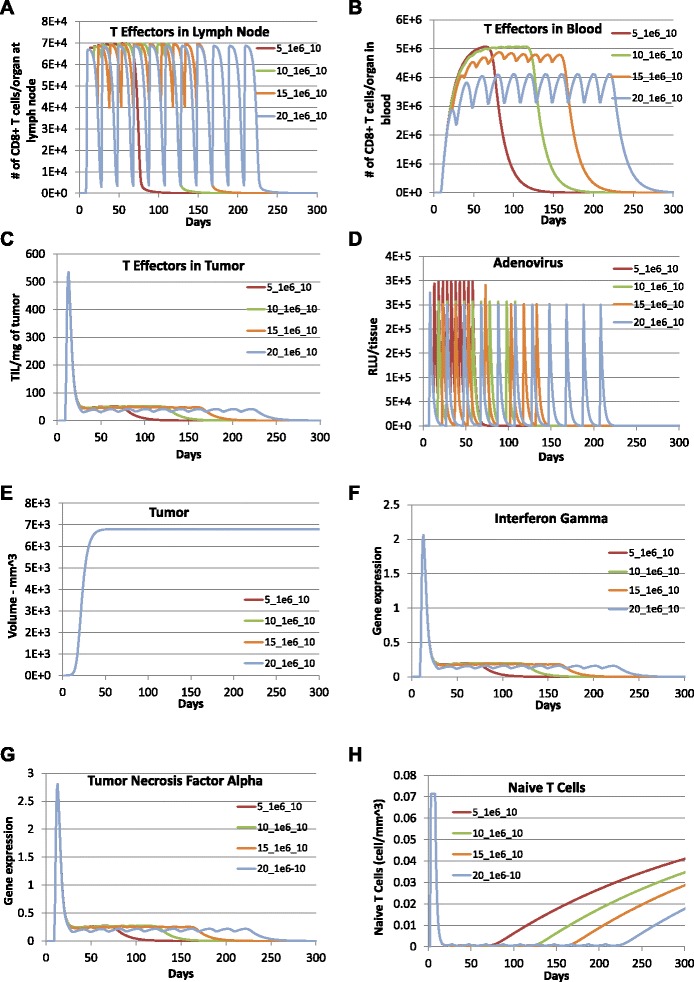
Prediction of effect of multiple vaccinations with increasing time between successive vaccinations. To examine the impact of the time between successive vaccinations, model predictions were obtained with adenovirus vaccination every 5 days (red lines), 10 days (aqua lines), 15 days (orange lines), 20 days (blue lines) with dose 1×10^6^ RLU per mm^3^ for 10 times following the initial vaccination with dose of 1.1×10^6^ RLU per mm^3^ on day 5 after tumor implantation. **a** TE1, **b** TE2, **c** TE3, **d** LV, **e** Total tumor cells (CMHCI+ + CMHCI-), **f** IFNG, **g** TNFA, and **h** TN

Increasing the time period between successive vaccinations was the next variable that we explored (see Fig. 4). While the dose and number of immunization were kept the same, the biological responses were simulated for adenovirus vaccinations that were administered every 5 days, 10 days, 15 days, and 20 days with a dose of 1×10^6^ RLU per mm^3^ for 10 times in total. Simulation results suggest that shorter time periods between successive immunizations generate higher maximum immunizations magnitudes. In addition, immunizations last longer with longer time periods between successive immunizations. Length of T cell responses in all compartments would increase with the increase in time periods between successive vaccinations and then drop to zero in about 60 to 100 days after the last vaccination for all four cases with a smaller duration of response for longer time between successive immunizations. In general, the increased time periods between successive immunizations result in a 4 *%* to 18 *%* decrease in the maximum magnitude for T cell concentration in blood and almost no impact on the maximum magnitudes of T cell concentrations in lymph node and tumor microenvironment. The expression of IFNG and TNF *α* do not seem to be affected although shorter time periods between successive immunizations correspond to shorter durations of both cytokine responses. In summary, simulation results suggest that with the same dosage and number of times of vaccinations, the shorter the time between successive vaccinations, the higher T cell concentration in blood and the shorter the T cell responses in all three compartments. However, the increase of time periods between successive vaccinations does not seem to affect tumor growth.

**Fig. 5:**
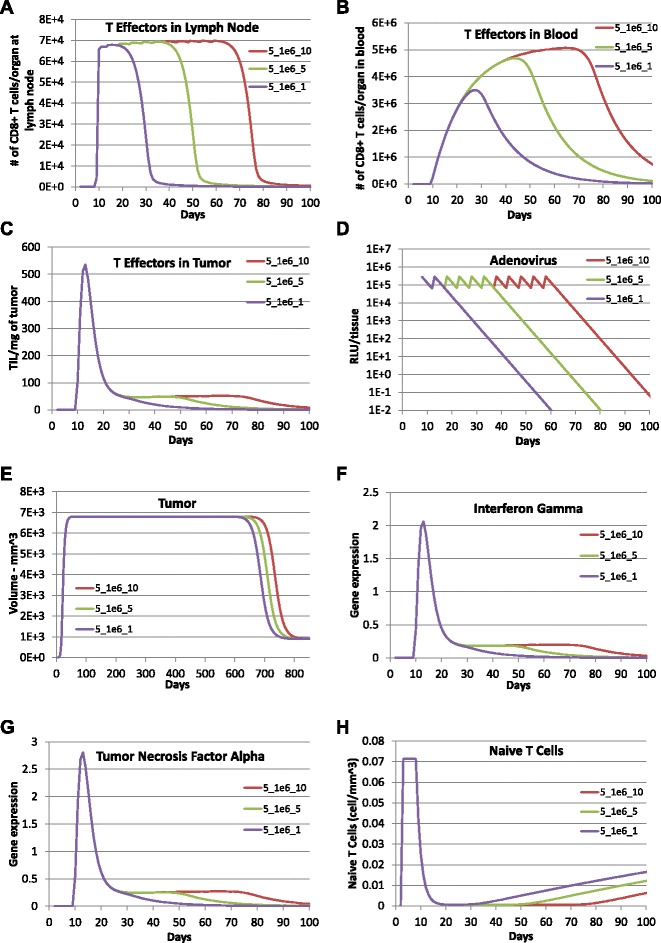
Prediction of effect of multiple vaccinations with enhanced T cell cytotoxic ability and saturation constant. The simulation results were obtained with enhanced T cell saturation constant *α*=7.82349×10^11^ and T cell cytotoxic ability *c*_4_=37350 and the rest of parameters as listed in Table 1. To examine the impact of the frequency of vaccination with enhanced T cell cytotoxic ability and saturation constant, model predictions were obtained with adenovirus vaccination with dose of 1×10^6^ RLU per mm^3^ every 5 days for 1 time (purple lines), 5 times (green lines), 10 times (red lines) post the one time vaccination on day 5 after tumor implantation with dosage 1×10^6^ RLU per mm^3^ in the experiment. **a** TE1, **b** TE2, **c** TE3, **d** LV, **e** Total tumor cells (CMHCI+ + CMHCI-), **f** IFNG, **g** TNFA, and **h** TN

We also explored the impact of increasing the number of times of immunizations with the same dosage and time period between successive vaccinations (see Fig. 5). Model predictions were obtained with adenovirus vaccinations with dose of 1×10^10^ RLU per mm^3^ every 30 days administered once, 5 times, and 10 times following the initial immunization. We found that, when dosage and time period between successive vaccinations were kept the same, increasing the number of times of immunizations increased the duration of the T cell. In addition, the increase in number of times of vaccinations only increased the maximum T cell concentration in blood by 8 *%* to 34 *%* but did not impact the maximum T cell concentrations in lymph node and tumor microenvironment. Figure 5 also shows an interesting result that the time period of the tumor staying in its maximum value and then drop to its carrying capacity is increased with the increase of number of times of multiple immunizations while increasing the length of the T cell immune response (i.e., $C_{\textit {MHCI}}^{+}\doteq 0$ and $C_{\textit {MHCI}}^{-}= \frac {k_{p2}-k_{d4}}{r_{2}}\doteq 1.497\times 10^{9}$ in number of tumor cells or equivalently, $s_{t}*C_{\textit {MHCI}}^{-}\doteq 898.2 \,mm^{3}$ in tumor size).

### Impact of the single adenovirus vaccination with enhanced T cell cytotoxicity or proliferation

**Fig. 6:**
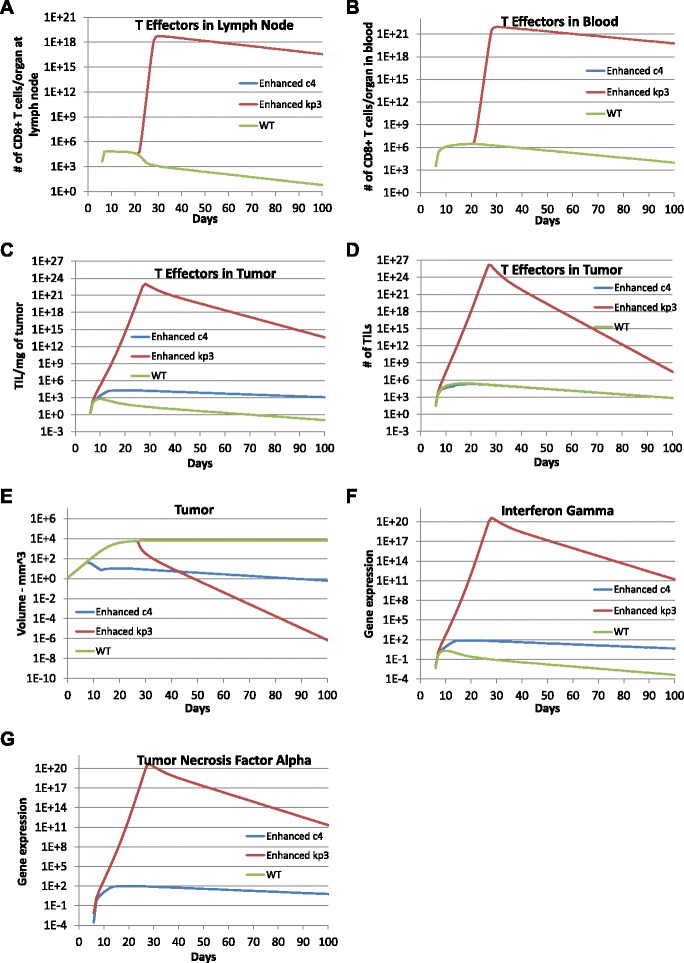
Improved one time vaccination effects with enhanced T cell cytotoxic ability or proliferation. Model predictions were obtained with enhanced T cell cytotoxic ability *c*_4_=2.49×10^5^ (blue lines) and increased T cell proliferation rate *k*_*p*3_=5.73 (red lines). Results were compared to the model prediction obtained using calibrated parameter values as listed in Table 1 (green lines) to explore the impact of a single vaccination on day 5 after tumor implantation with dose 1.100481×10^6^ RLU per mm^3^ with enhanced T cell cytotoxic ability or local T cell proliferation. **a** TE1, **b** TE2, **c** TE3 expressed as cells per mg tumor, **d** TE3 expressed as total cells, **e** Total tumor cells (CMHCI+ + CMHCI-), **f** IFNG, and **g** TNFA

Finally, we explored the impact of a single adenovirus vaccination with enhanced T cell cytolytic ability or T cell proliferation on tumor growth by changing the corresponding parameters. Model predictions with enhanced T cell cytolytic ability (i.e., *c*_4_=2.49×10^5^) and improved T cell proliferation rate in the tumor compartment (i.e., *k*_*p*3_=5.73) were obtained and compared to the prediction using calibrated parameters reported in Table 1 (see Fig. 6). The cell concentrations of naïve CD8 ^+^ T cells and adenovirus are not included in the figure as the model predictions were exactly the same for all three cases. With an enhanced T cell cytolytic ability, the concentrations of effector CD8 ^+^ T cells in the lymph node and blood as well as the total number of T cells in the tumor were almost the same relative to simulations using the calibrated parameter values. In contrast, the concentration of effector CD8 ^+^ T cells in the tumor, IFNG and TNF *α* were increased by 100 to 1000 times compared to the prediction obtained using the *c*_4_ from model calibration. The increase in CD8 ^+^ T cell response within the tumor decreased the tumor size to effectively zero (i.e., less than the size of a single tumor cell) within 100 days. To check whether tumor would relapse during the average life span (1 to 2 years) of a mouse, we have run the simulation for more than 800 days and found that tumor cells continued to decrease to near zero after 100 days as shown in Fig. 6. Collectively, the simulation results suggest that tumor cells can be completely eliminated by a single adenovirus vaccination with greatly strengthened T cell cytolytic ability (*c*_4_). In the model prediction with enhanced T cell proliferation rate *k*_*p*3_, we see that T cell concentrations in all three compartments were greatly enhanced and tumor cells were reduced to near zero within 100 days. To summarize, the simulation results suggest that the tumor may be completely eradicated by a single adenovirus vaccination by enhancing either the CD8 ^+^ T cell cytolytic ability or the CD8 ^+^ T cell proliferation rate.

## Discussion

Mathematical modeling and simulation are increasingly being used in the pharmaceutical industry to better understand the underlying biology targeted by a drug and to explore therapeutic scenarios that may be difficult to test experimentally [45]. Here, we developed a three-compartment mechanistic mathematical model to describe the clonal expansion of CD8 ^+^ T cells in a mouse model of metastatic melanoma in response to adenovirus vaccination against a defined tumor antigen. Based on the collective knowledge of this pre-clinical mouse model, the model represents the primary CD8 ^+^ T cell response to adenovirus immunization and the subsequent impact on the growth of a tumor derived from the B16F10 cell line. Using the mechanistic model as a framework to integrate different experimental studies, model parameters were calibrated against published experimental data that describes the primary response. As shown in Fig. 2, our model predictions of adenovirus concentration, tumor size, concentrations of CD8 ^+^ T effectors in blood and tumor, gene expression of IFNG and TNF *α* matched the experimental data. The proposed model structure reflects a trade-off between biological realism, parameter identifiability, and a fitness-for-purpose. As mentioned above, an excess of data points (93) relative to the number of parameters (27) suggests that the model is identifiable in theory. Efforts are underway to identify the appropriate topology of the network, given the available data [46].

In terms of the trade-off between biological realism and fitness-for-purpose, we settled on the proposed model structure to facilitate stability analysis. Stability analysis of tumor-free and high tumor equilibria was conducted based on the linearized system. Impulsive stabilization using the Lyapunov method will be considered in the future to provide conditions on parameters such that the high-tumor equilibrium may be stabilized using impulsive control through manipulation of strength and frequency of the multiple vaccinations. However, the proposed model structure imposes some limitations in how the model represents the system and interpreting the model predictions. In particular, we note that the model predicted an earlier peak time for the concentration of effector CD8 ^+^ T cells in the lymph node compared to experimental data, which may suggest a more complicated model structure for the lymph node compartment than proposed here. While additional model structure may help in capturing the dynamics of T cells within the lymph node, the current structure is sufficient to capture the dynamics of CD8 ^+^ T cells within the blood, which is the pool that gets recruited to the tumor compartment. Additional lymph node structure would then have limited impact on our conclusions. We also note that the effective concentration of effector CD8 ^+^ T cells within the tumor compartment (e.g., number of effector CD8 ^+^ T cells per weight of tumor) peaked at 10 days despite the blood population of CD8 ^+^ T cells peaking at day 20. As CD8 ^+^ T cell recruitment from the blood into the tumor compartment was assumed to be independent of tumor size and the parameter values suggest that proliferation of CD8 ^+^ T cells within the tumor was negligible, this decline in CD8 ^+^ T cell concentration was due to dilution of recruited effector CD8 ^+^ T cells into an exponentially growing tumor mass. While a direct measure of tumor infiltrating CD8 ^+^ lymphocytes was reported at a single time point, expression of IFNG and TNF *α* are implicit surrogate markers for CD8 ^+^ T cell infiltration as these two cytokines are directly proportional to the concentration of CD8 ^+^ T cells within the tumor compartment. Measuring the number of tumor infiltrating lymphocytes in this mouse model at additional time points would help confirm these assumptions. This would be interesting, as tumors, like the B16 model, are known to develop immunosuppressive mechanisms that are proportional to tumor size that could alter the relationship between the presence of tumor infiltrating lymphocytes and cytokine production [47].

Given the rapid growth of the B16F10 model and ethical limitations of animal studies, studies using this pre-clinical mouse model is limited typically to a single round of therapy. Yet, the treatment of human cancers typically involves multiple rounds of therapy to control tumor growth. Here we used a calibrated mechanistic model coupled with computer simulation to explore clinically relevant treatment options *in silico*. In exploring the impact of multiple vaccinations, our model indicates that increasing the dose of adenovirus vaccination, the time period between successive adenovirus vaccinations, or the number of adenovirus vaccinations results in a prolonged lifespan of effector CD8 + T cells in all three compartments and extended length of secretion of the cytokines IFNG and TNF *α* within the tumor microenvironment. However these changes in multiple vaccinations have little impact on the magnitude of the clonal CD8 ^+^ T cell immune response and therefore have very little impact on reducing tumor growth. If technically and ethically feasible, additional animal experiments using multiple vaccinations may be helpful to confirm these predictions. As the adenovirus vector promotes clonal expansion of CD8 ^+^ T cells that recognize a small number of epitopes derived from tumor antigens, the number and diversity of effector CD8 ^+^ T cells might not be sufficient to eliminate tumor cells completely. The results are consistent with recent findings in literature, where Budhu et al. reported that CD8 ^+^ T cell concentration determines their efficiency in killing melanoma cells [48]. As reported in [49, 50], immunotherapy of patients with cancer requires the in vivo generation of large numbers of highly reactive anti-tumor lymphocytes that are not restrained by normal tolerance mechanisms and are capable of sustaining immunity against solid tumors. Immunization of melanoma patients with a broader array of cancer antigens can increase the number of circulating effector CD8 ^+^ T cells (eCTLs), but to date this has not correlated with clinical tumor regression, suggesting a defect in function of the eCTLs. In contrast, a clinical benefit has been observed in patients with metastatic melanoma using antibodies against CTLA4, which globally increase the number of circulating CD8 ^+^ T cells irrespective of antigen specificity [4]. The one-time adenovirus immunization experimental data [15, 16] and the computational simulations exploring the efficacy of multiple adenovirus vaccinations using our calibrated model all indicate the limited impact of a single antigen-specific therapy to eliminate tumors. Our simulation results also suggest that increasing the cytotoxic activity of effector CD8 ^+^ T cell or the local proliferation of CD8 ^+^ T cells within the tumor microenvironment, as observed following anti-PD1 therapy [51], may completely eliminate tumor cells.

## Conclusions

In summary, we present a multi-scale mechanistic model of CD8 ^+^-mediated control of tumor growth in response to adenovirus-vaccination mediated T cell stimulation using a system of impulsive ordinary differential equations. The model parameters were calibrated against experimental data employing a genetic algorithm whose fitness function is given by a linear combination of sum of normalized error squared and sum of normalized difference of slopes squared. With the calibrated parameter values, our model predictions match experimental data very well. Stability analysis via linearization implies that, in the case of no vaccination treatment, a small tumor will grow to its carrying capacity as a result of a stable tumor-free equilibrium and a unstable high tumor equilibrium. Using the calibrated model, numerical simulation of multiple adenovirus vaccinations suggest that this treatment strategy will significantly prolong T cell immune response but not necessarily enhance a cytotoxic CD8 ^+^ T cell response to a tumor antigen that noticeably reduces tumor size. A reduction in tumor size can be obtained if the cytotoxic activity or proliferation of effector CD8 ^+^ T cells present within the tumor microenvironment are enhanced. Along those lines, simulation results also show that a tumor may be completely eliminated by a single adenovirus vaccination that creates a highly enhanced cytotoxic T cell efficacy or with enhanced local proliferation of cytotoxic T cells within the tumor. Overall, the results illustrate how mechanistic models can be used to predict tumor growth response to antigen-specific immunotherapies and screen in silico for optimal therapeutic dosage and timing in treating patients with cancer.

## Ethics

The authors declare that no animal or human experiments were performed as part of the research for this manuscript.
